# Practical Observations in Midwifery. Part the Second

**Published:** 1832-10-01

**Authors:** 


### Medico-Chirurgical Review,

### No. XXXIV.

JULY 1, TO OCTOBER 1, 1832.

### I.

**Practical Observations in Midwifery, with a Selection of Cases. Part II. By *John Ramsbotham*, M. D. &c. &c. Octavo, pp. 507. Highley, July, 1832.**

NEARLY ten years have elapsed since we presented our readers with an account of the first part of the work, whose completion is now before us. The delay, our author observes, need not cause regret in the mind of the reader, since it has enabled the author to verify, by a more extended observation, the correctness of those principles, on which the practice should be grounded in the different emergencies. The author has been gratified by the re-publication of his former work in America, under the superintendence of that experienced physician, Dr. Dewees, and, in gratitude, dedicates the present volume to him and to Sir C. M. Clarke. He informs us that his Observations are confined to practical points, and that he has abstained, as much as possible, from theoretical discussions. This, we think, is a wise course, more especially in midwifery matters. In imitation of our author, we shall proceed at once to give an analytical sketch of some of the chief practical points discussed in this volume, particularly the subjects of breech and shoulder presentations, uterine hæmorrhage, puerperal convulsions, twins, abortion, and retroversion of the uterus.

I. *Breech Presentation*. The average occurrence of this may be roughly calculated at 1 in 30, independently of those which are made breech presentations by artificial turning. There are no signs, during pregnancy, by which we can recognize this particular location of the child.

"The commencing symptoms of labour also resemble those under a natural case, but they frequently proceed more slowly; the uterine contractions being, on the onset, shorter and more distant, so that a slighter impression is made on the maternal passages in a given time, and the descent of the presenting part is more gradual. The os uteri relaxes and opens, through which the membranes protrude; their bag in due course gives way, and the liquor amnii is discharged. The breech is afterwards pushed down by the increased impulse of uterine action; during its descent, its several parts are naturally accommodated to the pelvic cavity; at length, whether the belly or the back of the child is looking towards the mother's spine, one of the nates, taking the precedence of its fellow and assuming a conical shape, begins to distend the external parts. After considerable extension, the breech makes its exit, with one hip inclined towards the pubes, with the other towards the anus. The legs are presently set at liberty, and the trunk is expelled with one side under the pubes; with the other in the sacrum." P. 6.

By examination per vaginam, the breech will be detected, even at or above the brim of the pelvis, by its softness and roundness—by the absence of the cranial resistance—and the inability to define the sutures; and, as labour advances, the anus and genital organs may be detected by the finger; and usually some meconial matter escapes with the other discharge from the passage. In the generality of cases, Nature, unassisted, and uninterfered with, will complete the delivery; it is only in expediting the expulsion of the head, in order to prevent any protracted pressure upon the umbilical cord, that the assistance of the accoucheur may be necessary; and his most important duty is to direct the position of the head, so that it most easily accommodates itself to the diameters of the pelvis;—when it is placed diagonally, or laterally at the outlet, we must carefully turn it somewhat round, till the face lies in the hollow of the sacrum. But occasionally the cause of the detention is at the brim; and then the following directions given by Dr. Ramsbotham must be attended to.

"If it should be found, as is frequently the case, that the long diameter of the head is opposed to the conjugate diameter of the pelvis, that the forehead or the occiput is directed to the projection of the sacrum, such position of the head must be changed for reasons too obvious to mention, and the face must be made to assume a lateral or a diagonal direction. In that direction, the head must be gradually and cautiously drawn through the upper strait. When it has cleared the upper strait, and has gained possession of the pelvic cavity, its relative position must be again changed, by the inclination of the face into the hollow of the sacrum." 11.

It is well known that the state of the circulation in the umbilical cord is to be the rule of our practice in expediting the delivery of the head, or not. Occasionally we observe, that when the body of the fœtus has been expelled, but the head still remains in the vagina, that the abdomen and thorax distinctly heave several times, indicative of an obstructed transmission of the blood, and of an attempt to establish the new function of respiration. Such cases require prompt release; else the child will be lost. We are glad to find that so experienced and talented a practitioner as our author strongly inculcates the advantages of not hastening by any manual attempts, or otherwise, the early stages of breech cases;—if slowly and gradually completed, the parts are sufficiently distended and relaxed by the protruding conical bag of membranes to permit the head, the most bulky part, to pass without much difficulty;—an impatience or officiousness are the worst enemies of speed in midwifery. Cases however occur which require the assistance of the bent finger or of a blunt hook being carried over one of the groins, with which traction downwards must be cautiously made; and if, unfortunately, such deformity exists, as to preclude the exit of the head, without considerable danger to the mother, we are obliged, after bringing the body and arms down, to perforate the cranium. With this intention, the nape of the neck must be brought close under the arch of the pubes, and two fingers being carried as high as possible against the occipital bone, to direct and guard the perforator, an opening must be made by a semi-rotatory motion of the instrument; the contents of the skull evacuated, and a crotchet, or blunt hook, be then inserted upon its base;—in withdrawing the disfigured head, it is a matter of great importance to accommodate its different dimensions to the capacities of the pelvis. Where improper force has been used in delivering a breech presentation, the body has been sometimes separated from the head, and this left behind in the uterus; to remove it, let the uterine tumour be steadied by an assistant; and having introduced the left hand within the womb, and placed the fingers against some part of the head, the point of the perforator must be cautiously directed to it, and the operation finished as usual.

In such difficult cases of breech delivery, the removal of the placenta is usually attended with some trouble. We shall illustrate the above remark, by transcribing a few of the judiciously selected cases from the present work, whose chief and distinguishing merit (and that in the present day of authorship is not a small one) consists in a faithful and well-told narrative of events, as they occurred at the bed-side;—and as the examples always bear the impress of unvarnished genuineness, the reader feels gratified and instructed by perusing them, and is led thereby to reflect how he himself should act in such and such emergencies. We shall not, however, here anticipate our judgment of Dr. Ramsbotham's book, or commend it too highly to our obstetrical readers, before we have conducted them through its contents, and displayed its stores before them.

*An ordinary difficult Breech Case.*

" At 10, p. m. August 13th, 1821, I was summoned by a note from one of the midwives of the Royal Maternity Charity, to attend a poor woman in the parish of St. Luke, Old Street, which stated, " that the woman was in labour of her first child with a breech presentation; that the scrotum of the boy was out of the external parts, and so swelled, that she was afraid it would burst; and that the vagina was very rigid and would not give way." Upon attending to this call, I found the case as above stated, and that no advance had taken place for many hours; the breech was certainly in the pelvis, yet not very low, although the scrotum was external. The general appearance of the woman, connected with the other circumstances of the case, induced me to attempt to get down a leg, by hooking a finger over the groin; but in that attempt, I was completely foiled. The failure therein arose partly from the breech being situated too high to allow by means of my finger a sufficient purchase, and partly from the parts being jammed at the brim of the pelvis. I, therefore, had recourse to the blunt hook, and having passed it over one of the groins of the child, I proceeded to make extraction; but I was obliged to use a very considerable share of force before I could procure any descent. By perseverance, however, I succeeded in extracting the breech; the rest of the child followed afterwards in the usual manner. The child was still-born.

The difficulty here arose from the impaction of the breech within the pelvic brim, which was narrow in its general dimensions." 29.

*A Case of Difficulty produced by improper Attempts at Extraction of a Breech Presentation.*

" At 6, a. m. Tuesday, March 28th, 1820, I was called in a hurry to the assistance of a lady in Broad Street, who had been some time in labour under a breech presentation. On my arrival at her bed side, I found the breech and trunk external as far as the axillæ; but the arms, shoulders, and head were in the pelvis, or above its brim, and all the efforts of her medical attendant had hitherto been unsuccessful in bringing them down. In this situation they had remained for more than an hour; of course the child was already deprived of life. Upon making an examination, I soon discovered the cause of failure in my predecessor's attempts. He had got the head locked in the pelvis with the occiput behind the pubes, and with the chin to the sacrum. By a little inclination of the trunk sideways, by which the relative situation of the shoulders and head was somewhat changed, after some trouble, I was enabled to release the arms by means of my fingers; afterwards changing the position of the face, I brought the head through the pelvic brim; but that act required the exertion of considerable force, as the projection of the sacrum was unusually prominent. Notwithstanding the difficulties attendant upon the delivery, the lady recovered without any subsequent inconvenience." 39.

*A very difficult Breech Case, from Deformity of the Pelvis.*

"About noon, Wednesday, November 9th, 1814, I was called to the assistance of Mrs. M. in Finsbury district, in labour of her first child, a woman of low stature, and about forty years of age. Her labour-pains had commenced on the evening of the Monday preceding, and soon afterwards her medical attendant was summoned, who had been with her the greater part of the intermediate time. On examination, I immediately detected the breech lying above the brim of the pelvis; which, as far as I could judge from the nicest measurement I could make, did not possess a space equal to two inches from pubis to sacrum. As the os uteri was but little dilated, and the woman's strength not much impaired, I merely recommended for the present an occasional enema. I visited this patient again at six in the evening; at this hour, the os uteri was become a little more open, as well as more flaccid; the labour-pains were more forcing; but no descent of the breech could take place for want of space. Seeing no possibility of improvement, I now determined upon immediate delivery, which I foresaw would be a task of no little difficulty. Having introduced my left hand within the vagina, I passed its fore-finger over one of the groins of the child, and upon it I insinuated a blunt hook, which gave me an excellent purchase. After exerting a good deal of force, I managed to get down the leg of that side. I then carried the blunt hook over the other groin, and got down the other limb in a similar manner. The possession of the legs enabled me to extract the trunk as far as the sxillæ. The arms were at this time drawn up on each side of the head; with some difficulty, I carried the blunt hook over one of these, which enabled me to bring it down. I proceeded in a similar manner with the other arm; but the most difficult part of the delivery I had yet to encounter in the extraction of the head; for it was impossible that the head could pass entire through such a pelvis, and it seemed to me to be no easy matter to perforate it. After a careful enquiry into its exact position, I brought the occiput close behind the pubes; then passing the two fore-fingers of my left hand against the under part of the occipital bone, with the perforator, I made a large and free opening through the skull into the brain. Within this opening I inserted the blunt hook, and getting thereby a very firm purchase upon the base of the skull, by the continuance of the extractile power it afforded, under the exertion of which the contents of the cranium were largely expelled, I succeeded in extracting the head. The placenta followed immediately. The next day the woman had procured refreshing sleep during the night; had passed urine; and, indeed, seemed as comfortable as if she had undergone no unusual inconvenience. She ultimately recovered as well as after the most natural labour, and is still living." 34.

Our author next leads us on to the consideration of shoulder-presentations, or, as they are vulgarly termed "cross-births." No satisfactory indication of such irregularity before labour begins is known; probably the abdomen may be broader from hip to hip than usual, with a diminution of extension upward; occasionally certain, not easily described feelings are experienced by the woman, which lead her to suspect something strange and uncommon; but assuredly she is much more frequently wrong than correct in such apprehensions and presentiments. The labour commences as in a natural case, but it proceeds very tediously; for although the pains may be vigorous and regular, little progress is made; the presenting part does not proportionally descend, but continues to occupy the brim of the pelvis:—even by manual examination it is not very distinctly detected at first, unless indeed an elbow or a hand have slipped well down. But the difficulty of the diagnosis must awaken the more attention and care, in order that, if possible, the nature of the case may be ascertained before the membranes burst; for no sooner do the waters escape, than the pains generally become more powerfully detrusive, and the parietes of the womb more closely embrace the body of the child;—to trust to Nature completing the process in this presentation, is to sacrifice the life of our patient; the cases being very rare, where, in consequence of the ample dimensions of the pelvis, and the comparatively small size of the child, this has been squeezed through with much difficulty, and after great suffering. The observations of the author we know to be so practically important, that we cannot do better than transcribe them.

"Every case, in which the presenting part does not readily come within the range of the finger, especially after the establishment of pains and relaxation of parts, ought to receive an unusual share of watchful attention. For that circumstance ought to excite a justifiable suspicion, that some other part of the child except the head, may be placed at the brim of the pelvis. And until that point is satisfactorily cleared up, the mind is kept in a state of anxious suspense, with regard to the subsequent management of the case. During this interval, it is a matter of some moment to preserve the bag of membranes unbroken, as long as possible. The bag may by accident be inadvertently ruptured under an examination; but to prevent that occurrence, the necessary enquiries may be made in the absence of uterine contraction, under a flaccid state of membrane; they ought not to be frequently repeated, at least for the present.

Under such a state of uncertainty, I consider it to be an indispensable duty on the part of the accoucheur, either to remain in the house of his patient, or to be within instant call. I have known a breach of attention to this important maxim to be followed by the most serious consequences. The membranes have suddenly given way during his absence; he has lost the momentary opportunity of delivering his patient by turning; the pains have become urgent and forcing before his arrival, and the woman has had subsequent difficulties and dangers to encounter, which timely aid would certainly have prevented." 45.

The importance of being able to ascertain the presentation of the child, before the rupture of the membranes, cannot be too urgently impressed; for by adroitness the accoucheur is sometimes able so to poise the child, still floating freely in the water, upon his finger, as to push up the projecting part, and thus bring the head round to the os uteri. That most dexterous obstetrical operator, Dr. Hamilton of Edinburgh, inculcates the possibility of this manœuvre in his lectures. We need not describe the process of turning, and shall satisfy ourselves by alluding to a very embarrassing difficulty which sometimes occurs. Suppose that one or both feet have been brought down, while the shoulder and chest are still protruding—the descent of the breech and trunk is thus prevented; and our object must be to push the shoulder out of the way, while at the same time we continue pulling the legs; but frequently it is exceedingly difficult to retain hold of them, partly in consequence of their being so slippery, and partly from the weariness, and numbness of the operator's hand. In such cases let a noose of strong tape be put round the ankle, by which traction may be made, while we endeavour to raise the obtruding shoulder;—a steady and cautious perseverance in this method will almost always enable us to succeed.

In those most difficult and dangerous cases, when the hand cannot safely be introduced into the uterus, from its closely grasping the fœtus, it is necessary either to separate the head from the trunk, by dividing the neck by means of a strong hook, having its internal surface ground to a cutting edge; or to perforate the chest, and to eviscerate its contents and those of the abdomen: the crotchet may be then applied within the pelvis, and the child gradually withdrawn. Dr. Ramsbotham decidedly objects to the practice of twisting, or cutting off the projecting arm; on the ground, that when this is done, the delivery will be very little facilitated, and such a confusion is produced within the vagina, as to make it almost impossible to discriminate the parts of the child from those of the mother. In shoulder-presentations, a change in the position of the child is sometimes, although very rarely brought about by the unassisted efforts of the uterus itself. The process was first noticed by the late Dr. Denman, and designated by him the "spontaneous evolution of the fœtus:" he does not appear, however, to have correctly understood the mode in which this was effected; and we are indebted to Dr. Douglas of Dublin for the most satisfactory explanation. The following description by our author conveys a very good idea of it.

"The arm, shoulder, and chest, are propelled downward by uterine action; the strength and continuance of which direct the apex of the shoulder under the arch of the pubis, while the chest and part of the trunk are occupying the cavity of the pelvis. During this time, the trunk is undergoing a considerable diminution in its original bulk by the bending of the back, and the doubling of the belly; there is also some change of position in the different parts of the child. These advantages permit the entrance of the breech at one side of the brim of the pelvis; then the continued action of the labour pains pushes that part lower and lower, till it gains possession of the hollow of the sacrum; after which, it is gradually expelled under an unusual degree of personal suffering, and of perinæal extension. Under this process, the life of the child is generally destroyed by the violence of the expulsive efforts." 58.

To illustrate the preceding remarks we shall select a few of the cases.

*A very Difficult Preternatural Case; the Shoulder Presenting.*

"About 10 at night, February 22d, 1809, I was called to the assistance of a respectable woman, in Fleet Street, the mother of several children, who had been in labour all the day under the care of her usual medical man. The membranes had given way about four in the afternoon, when a hand came down; upon which an attempt was made to turn the child which proved unsuccessful; and similar attempts had been made in the interval. A considerable time had been wasted in quest of a particular accoucheur, who could not be found; and between nine and ten, the husband came to my house. On an examination, I detected the shoulder at the brim of the pelvis; both hands, with the funis without pulsation, down in the vagina; the uterus firmly contracted upon the child, and the pains violent and expulsive. I made an attempt to introduce my hand within the uterus, and after some perseverance, I did partially succeed, yet not to that extent as to command a foot; but, after some time, my hand became so cramped, and so completely useless, that I was obliged to withdraw it. I now thought of decapitation, but how to accomplish that act, I at that time scarcely knew. The late Dr. Combe had shewn me, a short time before, a pair of decapitating scissars, which he considered well adapted for that purpose; I procured that instrument, but I found it in my hands entirely useless; being unable to surround the neck of the child. We then determined to call in Dr. Combe himself, under the idea, that he might use his own instrument more dexterously. Upon his arrival about midnight, he made an attempt to decapitate, but was also foiled. Upon this Dr. Combe introduced his hand partially within the uterus, and after much perseverance, he got a finger over one of the hams, and fixing there a blunt hook, he brought down a foot to the brim of the pelvis; being now sufficiently tired, he returned the management of the case to me. Seizing this foot I gradually got down the leg; and making my purchase downward by the leg, at the same time, with my other hand pushing up the shoulder, I at length succeeded in bringing down the breech, and in finishing the delivery. Doctor Combe was nearly two hours in effecting the first part of the operation, yet there was no appearance of blood, and the pains were strong throughout. The next day this woman, notwithstanding the length and degree of her sufferings, appeared uncommonly well, and recovered as soon as after any of her previous labours. 68.

It appears that it has been only within the last 12 or 14 years that Dr. Ramsbotham has been in the habit of perforating the chest in difficult cases of shoulder presentation. Previous to that time he was not acquainted with the practice, and resorted either to turning, although that operation was frequently very dangerous to the mother, or, in extreme instances, to decollation. He is now quite satisfied of the superior facility and safety of diminishing the bulk of the projecting part by opening the thorax, and discharging its contents. We may have less scruple to follow his example, as the life of the child is very rarely saved under any practice. Should any deformity of the pelvis exist, so as to prevent the easy expulsion of the head, it may be necessary to perforate it under the occipital bone, and afterwards, by the assistance of the crotchet, to finish the delivery. The following case is a very valuable one, and although very lengthy in narration, is worthy of study by all.

" *A Shoulder Presentation under a high degree of Uterine Contraction, delivered by Perforation of the Chest, and subsequent Decollation.*

" Mrs. D. fell into labour of her second child in the early part of the day of Wesnesday, September 29th, 1819. Her former labour had been slow and protracted, but it was at length safely terminated by the natural agents, under the care of a respectable midwife. For her present confinement, she determined to engage a male practitioner, and unfortunately selected one, whom the sequel will shew to have been quite undeserving of her confidence. The doctor (as he was termed) was soon informed of the commencement of her labour, and paid her a visit; but as the pains seemed then trifling, and as he lived in the neighbourhood, he did not remain long in the house, but went about his regular business. Some time after his departure, the membranes broke, and he was recalled about one o'clock in the day. Upon his return, he found a hand down in the vagina, with the shoulder at the brim of the pelvis; he made an immediate attempt to turn the child, but in this attempt he proved unsuccessful; attributing his failure to strong uterine action, and to want of room at the brim of the pelvis. After the lapse of a short time, he made a second attempt, in which he was also foiled. He now gave the woman a large dose of laudanum, and while she was supposed to be under its influence, this active practitioner made a third essay, which proved equally unsuccessful as the preceding ones. Not at all daunted by these repeated failures, and apparently determined to effect his object, towards night he repeated the dose of laudanum, after which he made several more ineffectual trials during the night, and even to the time now to be mentioned; a little before which, he confidently asserted that the child was coming. About five o'clock on the Thursday morning, the husband called me out of bed, and requested my immediate attendance upon his wife in a case of difficult labour. From his imperfect statement of the facts even, I was led to suspect, that I should have some difficulties to encounter in delivering the woman; but little did I then suppose they would have proved so formidable as I really found them.

Upon my entrance into the lying-in room, the poor woman, under a state of considerable exhaustion, bitterly bewailed the severe sufferings to which she had been obliged to submit for more than *sixteen* hours; and at the same time pitifully implored my assistance in her behalf. I found the left hand quite external, swelled, and discoloured; the arm in the vagina highly tumefied; the shoulder and part of the chest firmly impacted at the brim, and in the upper part of the pelvis, which was somewhat deficient in the usual room at the promontory of the sacrum; and the woman's parts swollen and tender. Besides, the uterus was so entirely embracing the child, that a finger could with difficulty be inserted between the two. The uterine tumor was extremely tender under the hand, comparatively small and firm; there was still an occasional tendency to uterine action, but in a slight degree. In the early part of the labour, the pains had been unusually strong and active; at this time, that activity was much diminished; and the constitutional symptoms were beginning to be alarming. Under these discouraging appearances, I could offer little hope of affording such assistance as would be the means of preserving the woman's life; as for that of the child, it was already gone.

The high degree of tonic contraction, which the uterus had already acquired, convinced me of the impropriety of any further attempts to deliver by turning; nay, even of the impossibility of the act, without the exertion of such violence as would endanger the structures engaged therein. Having determined that point, I had to devise some other mode, by which delivery might be accomplished; and decollation, or perforation of the chest seemed to me the only means of effecting that object. But before I had recourse to either of these unusual modes of practice, I was desirous of procuring the sanction of some other experienced practitioner. I therefore appealed to a highly respected friend, who readily favoured me with his presence, accompanied by my son. When that gentleman had satisfied himself of the facts of the case, he agreed in opinion with me, upon the impropriety of attempting to deliver by turning; we therefore determined upon perforating the chest; eviscerating the thoracic and abdominal cavities; and afterwards taking the chance of consequences. Accordingly about seven in the morning, with a large-sized perforator directed on my left hand, I made a free opening into the chest of sufficient capacity to admit the ready introduction of the hand, and proceeded to break down and extract the contents of the thorax. Having withdrawn such of these as I was able to get away, I passed the perforator through the diaphragm, and proceeded in a similar manner to abstract the abdominal contents. This part of the operation was attended with considerable embarrassment and difficulty; partly produced by the tenacity of the different viscera, and partly by the contraction of the original aperture. After being thus occupied for a length of time, I became fatigued, and requested my friend to relieve me. In his endeavours to withdraw the abdominal contents, he met with some part which gave unusual resistence. In his attempts to overcome it, he so far altered the position of the child, that the other arm came down into the vagina. Having now possession of both arms, by which the position of the diminished trunk could be somewhat regulated, I was enabled to surround the neck with my finger, over which I carried a common blunt hook, and by its side I passed my decapitator, which was easily brought through the neck. The headless trunk was now brought down by the arms, and the rest of the child followed. The head was withdrawn by the assistance of a blunt hook inserted into the mouth; after which the placenta was found to be naturally separated." 78.

This woman had four children after this very severe delivery; in the last, the shoulder presented, with the arm down in the vagina; the membranes had already given way, but by immediately turning, a living child was brought into the world. It is only when the shoulder and chest are found wedged in the brim of the pelvis, and the child closely embraced by the contraced uterus, that, after attempting to introduce the hand for the purpose of turning the child, but finding the attempt fruitless, that we should proceed either to decapitation, or to the perforation of the thorax and abdomen. In selecting the one or other of these proceedings, we must be guided by the position of the fœtus; whichever may be most easily effected, ought to be preferred. In either case, it is of great importance to encourage uterine action at the time when we are withdrawing the child. Many cases are reported in Dr. Ramsbotham's book, in which much time has been unnecessarily lost, and much pain inflicted by repeated attempts to turn, although the hand could not be passed between the shoulders and the uterine parietes; the strength of the patient may thus become exhausted, and life be lost if further delay is permitted. Let us select an example of the dangers of the ancient practice.

"*A Shoulder Presentation under a deformed Pelvis, in the Management of which the Head was separated from the Trunk.*

About noon, on the 1st of May, 1796, I was sent for to a young woman, who had been in labour of her first child more than twenty-four hours, under the care of a midwife. On making an examination, I felt an arm down in the vagina, with the shoulder at the brim of the pelvis; on a closer enquiry, I detected a very deformed pelvis; I likewise found, that the uterus was firmly contracted on the body of the child. Not liking to interfere in a case in which such difficulties presented themselves, without the presence of another practitioner, I called to my assistance a medical friend. Upon his arrival, he commenced an attempt to turn the child; after great exertions and the lapse of more than half an hour, he so far succeeded as to bring down a leg. By the assistance of its purchase, he was enabled, after some further time, to bring down the trunk, and afterwards to release the arms. An attempt was now made to extract the head; but in the application of the force requisite to effect that object, the head was separated from the trunk, and left within the uterus. It now became necessary to lessen the head by perforation, after which it was with difficulty extracted. This unpleasant operation, from its commencement to its conclusion, continued more than four hours; so that the woman appeared afterwards much exhausted, and gave little hope of recovery.

The next day, however, she had considerably rallied, and continued to improve for a few days. But it was now found that the vagina was in a state of sloughing. Under an improved regimen and the use of tonics, the slough in due time separated, and the woman ultimately recovered." 92.

We have already alluded to the practice of dismembering the projecting extremities; the following is an example in which success attended.

"This lady began to shew symptoms of commencing labour in the afternoon of Tuesday, and in the evening sent for him to her assistance. After he had been some time in the house, he made an examination, and found a hand down in the vagina, with the shoulder at the brim of the pelvis. He attempted to introduce his hand for the purpose of turning the child, but was foiled in that attempt. A neighbouring friend was summoned to his assistance, who also endeavoured to turn the child, but he was equally unsuccessful. The husband was then dispatched to my house at the hour above-mentioned; but in consequence of my absence from home, reference was made to another accoucheur. This gentleman also made an attempt to turn the child, which proved as futile as those of his predecessors. After consulting upon the state of the patient, it was at length agreed, to separate the protruded arm at the shoulder; and this suggestion was with considerable difficulty carried into effect. Soon afterwards the other arm made its appearance in the vagina, and was also removed. The principal agent in the operation, even after this mutilation, was still unable to accomplish his intended object of turning; he found it yet impossible to push up the protruded chest, and to pass his hand sufficiently high to reach the feet. About six in the morning, a full dose of an opiate was exhibited; and, after waiting its effects a few hours, a most unaccountable change was found to have taken place in the position of the child; for the head was then presenting at the brim of the pelvis. The head was lessened, and the child was extracted in the usual manner. After the delivery of the child, this lady seemed to require the exhibition of some stimulant. Upon her head being raised from the pillow for the purpose of giving her some brandy and water, she was seized with a convulsion fit, which suddenly carried her off." 98.

An interesting case is detailed, in which the woman had been in labour, under a shoulder-presentation, for 36 hours; and various unsuccessful attempts at delivery had been made by her medical attendant before Dr. Ramsbotham was summoned. He readily and easily turned the child, but the patient died on the second day; and dissection revealed a laceration of the structure of the uterus, just above the pubes. Whether this accident had been produced by the efforts previously made, or took place during the delivery, was not very obvious; the Doctor states that he was unconscious of much resistance to the passage of his hand. It is dangerous to persist too obstinately in endeavouring to turn, when the child is firmly impacted, as either the womb may probably be lacerated, or irreparable mischief inflicted on the soft parts. In cases of strong tonic contractions of the uterus, the child must almost necessarily be born dead; and hence the propriety of not delaying delivery by perforation too long.

One case more on the subject of shoulder-presentations, and we must proceed to other topics. It is one of spontaneous evolution, or natural turning. The detail is given in the words of the midwife who attended.

"I was called to Mrs. S. at four in the afternoon, and found her flooding; I gave her the acid drops, which had the desired effect. At eight in the evening labour came on, but the os uteri was high and rigid. The membranes formed largely, and I could have no idea what the presentation was; I, therefore, deemed it most prudent to let them rupture of their own accord; and to my consternation, the right arm presented. I wrote for the doctor; the pains became so strong, that the hand was soon through the externals. I entreated the woman not to bear her pains down, and I endeavoured to keep the arm back, but in vain. The shoulder passed the pubes to the externals; and the perinæum began to be protruded so very largely, that I was obliged to direct my attention to it. The side began to advance with the hip, the breech, and the legs, and lastly, the head. The child was still-born; the mother is doing well." 105.

Having now discussed the very important subject of præternatural labours, our attention is next drawn to one full of momentous interest, from its frequency and great danger.

There is no situation in which a medical man can be placed, more awfully responsible, than when summoned to attend a case of uterine hæmorrhage. The lives of two beings depend so often on his individual skill, that it argues not only the most disgraceful ignorance, but, at the same time, the most criminal apathy, if he neglects to make himself thoroughly acquainted with the treatment of a case which is so thoroughly important; what, then, shall we say of the judgment and good feeling of those who have dared to disparage the practice of midwifery, and have impudently denounced it as unworthy a professional man? In addition to its urgent practical moment, many are the opportunities which the accoucheur has of studying some of the most curious and interesting phenomena of life, in health and in disease. For example, where shall we see a more beautifully-devised adaptation of structure to function than in the anatomy of the uterus, and the curious provisions which are made for the arresting of hæmorrhage from its blood-vessels. We hope very soon to allude to this subject more particularly, in our notice of Dr. Robert Lee's essay on the Placenta, in which he has published the dissections by that very able anatomist, Mr. Owen, of the College of Surgeons; suffice it to say, at present, that it is only by the contraction of the fibres of the womb, that all hæmorrhages from its cavity can be effectually restrained; and that, therefore, the great aim and object of a medical man must be to induce this state. Coagula may plug the open vessels for a time; but, if we should ever trust to this uncertain protection, the life of our patient is held by a frailer tenure than was the sword suspended over the head of Damocles. As a general fact, we may affirm that, during the whole term of utero-gestation, any considerable discharge of blood from the vagina can only arise from the detachment of a portion of the placenta from its adhesion to the uterine surface. Occasionally, though rarely, when the accident occurs in the early months of pregnancy, the blood is retained in part within the womb, and has been voided, one or more months after, as a firm flattened substance, somewhat like a piece of half-tanned sole-leather. The farther that the pregnancy is advanced, the more alarming, cæteris paribus, will probably be the hæmorrhage, for the vessels become proportionally more and more capacious as the size of the uterus increases. In treating of this subject, we shall consider, first, those cases in which the bleeding proceeds from the detachment of the placenta, while this vascular mass is affixed to the fundus, or to the walls of the uterus; and, secondly, those in which it is situated partially or entirely over the mouth of this organ; to the former, Dr. Rigby, in his excellent essay, applied the epithet "accidental"—to the latter, "unavoidable." Now it is only by vaginal examination that we can obtain a correct knowledge of the fact; if the finger detects the membranous bag, with the presenting part within it, the case is at once determined to be one of "*accidental*" hæmorrhage; but if it encounters a fibrous flesh-like mass, adherent round the inner edge of the os uteri; and especially if, when the finger is pushed upward with a slight force, the bleeding is temporarily increased, the more serious accident is to be apprehended. A large coagulum, indeed, formed within, or at the os uteri, may possibly be mistaken for the placenta; but its surface will be found to be smooth and even to the touch, to possess a less degree of resistance than the placenta, and to be more easily penetrated by the finger, or it may be made altogether to recede. The treatment of accidental flooding consists in recommending perfect quietude in the recumbent posture; the use of cold drinks, ices, acids, and opiates; the application of cold to the abdomen; and sometimes the employment of the plug. Should these means fail, Dr. Ramsbotham very judiciously urges the propriety of rupturing the membranes, and discharging the liquor amnii, whereby the womb is generally induced to contract, and thus to arrest the hæmorrhage. Extreme cases sometimes occur, when we are obliged to empty the uterus of its contents by turning: fortunately, the necessity for this step is rare; but if the loss of blood continues, after the escape of the waters, so as to endanger the mother's life, we have no alternative.

In performing this operation, the accoucheur will do well to attend to the following admirable precepts.

"Artificial delivery, especially when it is to be effected by the introduction of the hand, and by turning the child, can never be successfully attempted under a state of positive syncope, or of very great exhaustion. Under either of these states, the powers of the constitution seem unable to bear up against the shock occasioned by the additional pain and violence inflicted thereby. It is, therefore, highly desirable that a truce from the discharge should be previously obtained, if possible, by some means or other, and under such circumstances, the *plug* may sometimes be advantageously applied. The equilibrium of the circulation is in some degree restored by a little delay, and the functions of the arterial system are resumed. When such advantages are gained, delivery may be effected with less danger. Let it be remembered, however, that the object of a forced delivery is not the mere extraction of the child; the evacuation of the uterine contents; but that such extraction and evacuation may become the means of inducing uterine contraction, and through it, the cessation of the hæmorrhage by the constriction of the bleeding vessels. The extraction of the child should therefore be made in a slow and gradual manner, and due attention should at the same time be paid to the degree of contractile effort in the uterine parietes. If it be made too quickly, the uterus is so suddenly emptied, that the diminution of its size cannot keep pace with the rapidity with which the child is withdrawn; its parietes are therefore left under a state of irregular contraction or of flaccidity. Indeed, there are few situations, in which a parturient woman can be placed, more replete with danger, than that in which the uterine parietes, either in the act of turning, or after the birth of the child, are felt lax, unresisting, and wrapping around the hand like a piece of wet wash-leather. Instrumental delivery ought to be conducted in the same deliberate and cautious manner, with a similar reference to the above principle." 139.

In almost all cases, in which the child is delivered by manual interference, it is better to remove the placenta rather early after the birth, than to defer that duty for a length of time. It is well known that many have lauded very highly the efficacy of the secale cornutum in uterine hæmorrhage. Dr. R. has not employed it extensively enough, to warrant him in giving a decided opinion. In one case, he states, its effects in increasing uterine action appeared evident and satisfactory. In the narration of cases to illustrate the above remarks, we shall select only such as present some interesting peculiarities.

"Case CXXXI. *Expulsion of the Placenta before the Child.*"

"I found a woman between thirty and forty years of age, pregnant of her eleventh child, suffering under the usual symptoms of a sudden and violent loss of blood. The left arm was down in the vagina; the shoulder was pushed into the brim of the pelvis; and the pains were active. With some difficulty, I introduced my hand, got hold of the feet, and brought down the breech; but in turning the child, to my surprise and alarm, the placenta escaped externally before the breech was brought down. This circumstance induced me to hasten the extraction of the trunk, shoulders, and head; and fortunately my efforts were materially assisted by uterine contraction, which continued strong and effective. During this operation, there was no material increase of hæmorrhage. After the delivery of the child, the uterus felt firmly contracted, but the woman was under a state of great exhaustion; from which she was endeavoured to be roused by the free use of stimulants. But every means devised for her relief proved of no avail. In a few hours she became excessively restless, with difficult respiration, and did not survive the middle of the day.

In this instance, the placenta was largely separated from the uterus at the commencement of the flooding; so much so as very much to incommode the action of my hand in search of the feet; it seems to have been afterwards entirely detached by the act of turning. One essential point in the case deserving attention is, that after the escape of the placenta, there was no increase of hæmorrhage." 160.

After a sudden and profuse hæmorrhage from the uterus, a serous coloured discharge frequently occurs; and has been mistaken for the liquor amnii. The practitioner ought to be aware of this, and by careful examination he may always ascertain whether the bag of membranes is broken, or not. And here we again entreat our professional brethren to study most attentively every topic which has any reference to the treatment of flooding; their own peace of mind, and the happiness of the dearest relationships, are interested in their skill.

When the placenta is implanted over the os uteri, we have already explained that hæmorrhage is unavoidable: in such cases, the accident is rarely met with before the 6th month; it usually occurs between the 7th and 8th months, and is sometimes deferred to the middle, or even to the very end of the 9th month. The necessity of a speedy and correct examination is of the most urgent importance; for when the nature of the case is well ascertained, there is only one course which can be followed; viz. that of resolutely but cautiously turning the child, and withdrawing it. The chief point of consideration is to determine the exact time at which the operation ought to be done. As a general rule, the os uteri should be already well dilated, or at least so far relaxed, as to permit the introduction of the hand without much difficulty, before the delivery is attempted. No attempt should be made, forcibly to dilate it, by passing two or more fingers within its orifice, unless we intend immediately to introduce the whole hand. The directions given by the author are so judicious that we shall lay them unabridged before the reader.

"But in awaiting those changes which may ensure the performance of that operative act with the greatest ease and safety, the quantity of the discharge, and the mode in which it flows, should be carefully watched, as well as the symptoms which from hour to hour are induced upon the system. Let us beware, however, of protracting the delivery beyond that period, at which its intended object, the preservation of the patient, may be defeated. When the continuance of discharge has produced faintness, pallor of countenance, coldness of the extremities, and symptoms of that description, every minute's delay increases the degree of hazard to the woman's life. Upon the whole, it is better to have recourse to delivery an hour too soon, than an hour too late." 179.

In describing the steps of turning in such a case, Dr. Ramsbotham says, that at the moment of dilating and passing the os uteri, the hæmorrhage is tremendously increased, and if, at this moment, from alarm, or other cause, the operator should be induced to withdraw his hand, the consequences will be frightful.

The remark, although generally, is not uniformly correct. A case very lately occurred in our own practice, in which, when summoned, we found that already between seven and eight pounds of blood had been lost, and that it still flowed; but from the moment of introducing the hand, and beginning to dilate the os uteri, scarcely a spoonful of blood escaped. We have heard a similar observation made by that very experienced accoucheur, Dr. David Davis. Should the patient be in a state of great collapse and exhaustion, when we are summoned to her relief, our first object should be to try to revive her strength by appropriate cordials and stimulants, before we proceed to deliver by turning. Dr. Ramsbotham proposes, in some cases, to perforate the bag of membranes by a trocar pushed through the substance of the placenta, in order that contraction of the uterus may be temporarily induced. We shall permit the author to speak for himself.

"When I have been obliged to have recourse to a forced delivery by turning, under a state of great exhaustion, I have frequently fancied, that the shock inflicted upon the nervous system by the violence of the operation has greatly increased the danger of the woman, and has sometimes induced a fatal result. In reflecting upon this presumption, in case of sudden depression under a placental presentation, it has seemed to me desirable, if possible, to obtain a truce from the flooding before delivery is attempted, that the system may somewhat rally from its preceding effects. I have therefore thought, that if, in these desperate cases, by any gentle means, the liquor amnii could be discharged, without inducing a greater degree of placental separation, some advantage would be derived from uterine contraction, and the violence of the discharge would be thereby checked. I must however in candour declare, that I have not had an opportunity of realizing the practical effect of this suggestion, since it occurred to my mind; I offer it therefore merely as an object of future consideration. The method I propose is, to penetrate the centre of the placenta by a perforator, or other sharply-pointed instrument, and allow the liquor amnii to run off. If the discharge be thereby checked, delivery may be put off for a short time; but if the discharge should continue afterwards, delivery must not be delayed. Let it be clearly understood, however, that this act will not supersede the necessity of delivery sooner or later, and that it will cause some loss of the child's blood from the placental vessels.

I have repeatedly remarked, that among those cases which have terminated fatally, in several of them that event has seemed to me to be hastened by too quick an extraction of the child, and the too sudden evacuation of the uterus. If the hand in turning be allowed to enter the uterus without resistance, and if, after it is in complete possession of the cavity, no contractile effort be perceptible in the parietes, the extraction of the child should be gradually performed." 189.

In some very rare cases the placenta has been completely detached from the os uteri, and been expelled either into the vagina, or even through the external parts, and the dead child has afterwards followed, without any return of flooding, or further hazard to the mother. Dr. Ramsbotham alludes to six such cases, which have come to his knowledge; and in every one of them, the mother recovered; hence we may infer, "that less danger attends an entire, but natural detachment of the placenta, in these cases, than is consequent upon a partial separation of that mass; and that the expulsion of the child may afterwards be safely entrusted to the natural powers without further interference." We shall extract one example of this rare occurrence.

"About half after six in the morning of April 29th 1818, a messenger arrived at my house, sent by two medical gentlemen in attendance upon a lady at Upper Clapton, with a note to this purport. 'We are in attendance upon Mrs. H. whose situation is involved in great uncertainty from a placental presentation; the bleeding is going on pretty actively, and we wish for your immediate opinion.' On my arrival at the house of the lady, about eight, I was told by one of the gentlemen, 'that since the note was sent off, some strong expulsive pains had come on, which had expelled the placenta through the external parts before the head of the child, and that it was lying upon the bed. That before this occurrence, the hæmorrhage had been violent, yet not to that extent as apparently to endanger the woman's life; but that since the appearance of the placenta, the flooding had very much abated.' During our conversation on this unusual occurrence, the gentleman more immediately in attendance, who, at my arrival was in the bed-room of his patient, came down stairs, and reported, 'that the head was presenting at the brim of the pelvis, with a hand down by its side; that there was no want of uterine action; that the flooding had ceased; and that his patient did not seem much exhausted.' An appeal was now made to my opinion, as to the further management of the case; to which I replied, 'that as the flooding (the most dangerous symptom) had abated; as the labour-pains continued active; and especially as the woman's strength kept up, there did not appear to me an immediate necessity for a recourse to any means for hastening delivery; watch your patient for a short time, and wait the result; if the flooding should return, or if any dangerous symptom make its appearance, let us know.' In about half an hour after this interview, the gentleman returned with a cheerful countenance, and stated, that the child was expelled without further loss of blood, and that his patient was promising to do extremely well." 230.

As it is of the utmost consequence that the management of placental presentations be thoroughly understood, we shall no doubt please many of our readers by transcribing an illustrative case.

"I was requested to visit a lady of middle age, the mother of several children, at a short distance from London, under uterine hæmorrhage, in the last month of pregnancy. She had previously suffered under several similar attacks without pain or other inconvenience; which had generally come on suddenly, and after a short continuance had gradually ceased. A repetition of the flooding had occurred the preceding evening in an increased degree, which had induced her medical attendant to sleep in the house; yet during the night, there had been a mere weeping from the vagina, scarcely coloured. At the time of my visit, this lady was sitting up; she had a cheerful countenance, a firm good pulse, and seemed in tolerable spirits. Indeed her appearance could not have excited the least alarm in the most timorous mind, if the previous and repeated attacks of discharge had not induced a suspicion, that the placenta might possibly be placed over, or near the os uteri. There was at this time very little discharge, and no pain. After being in the house an hour or two, I was permitted to make a vaginal enquiry, but the result was not very satisfactory. For the os uteri was rigid, and but slightly opened; and at the extremity of my finger, I thought that

I could detect the placenta. Throughout the day, the discharge was trifling; in the evening the lady was in good spirits; little alteration had taken place in the state of parts; and I was requested to sleep in the house. Between one and two on the Monday morning, I was disturbed by the nurse, who came to tell me, that the flooding had returned very violently, and that her mistress had slight pains; I now found the os uteri somewhat more relaxed with a great increase of discharge; and in a very short time, faintness came on, with less firmness of pulse. Under this state delivery was proposed, to which the lady readily assented. In passing my hand through the os uteri, I necessarily separated a larger portion of the placenta, and now the flooding became excessive, so that syncope ensued. Upon entering the uterus, I had some difficulty in rupturing the membranes; the bag was so flaccid as to offer little resistance, and the uterus was very inactive. Seizing a foot as quickly as I could, I brought down the breech; but the separated portion of the placenta escaped down before it. Recourse was now had to the exhibition of brandy pretty freely. Placing my right hand upon the uterine tumour, I assisted the trifling degree of contractile effort, which it exhibited, by external pressure, while, with the left, I made a slow extraction of the child by the feet. Upon the passage of the head, a large quantity of liquor amnii mixed with blood instantly escaped. The placenta was now thrown down into the vagina. As the uterus remained large, and very imperfectly contracted, with a continuance of flooding, I passed my hand without loss of time within its cavity, the stimulus of which induced some contraction. Our patient was now in a state of syncope, from which she was somewhat recovered for a short time by another recourse to brandy, so that I had hopes that she would have rallied. In about an hour, however, she became extremely restless, and the powers of life continuing to decline, she expired within two hours after delivery. The child was still-born." 204.

The third section of the present volume is devoted to the illustration of puerperal convulsions, one of the most frightful accidents which occur in medical practice. The tossing of the limbs, distortion of the face, the starting, staring eyes, the clenched teeth, the agitated breathing, and violent throbbing of the heart, are the leading symptoms; the duration of the fit is not long; an interval of quiet follows, to be quickly succeeded by other paroxysms of spasm, and other intervals of repose, until either relief is procured by means of art, or the powers of the system are exhausted by the numerous repetitions. While the fit lasts, the patient is quite insensible. A considerable resemblance exists between an attack of epilepsy, and of parturient convulsions; but they are generically distinguished by the extreme rapidity of the course of the latter; and by the absence of all aura epileptica. The predisponent causes are singularly obscure. Dr. Ramsbotham has frequently remarked, that, as with other diseases, several cases have occurred soon after each other. Dissection has hitherto thrown very uncertain light on the pathology; little or no change in the cerebral structure being generally found. In some cases, the vessels of the meninges are surcharged with blood, while those of the substance of the brain have appeared almost exsanguined; in others, a breach of vascular structure within the head has been detailed; and effused blood has been discovered in the ventricles, partly in a fluid, partly in a coagulated state. Puerperal convulsions may come on either before, during, or after parturition; they threaten great danger both to mother and child; and if unrelieved by art, almost uniformly have a fatal result. The attack is sometimes most sudden and instantaneous; at other times, it is preceded by symptoms indicative of cerebral plethora; pains in the head, confusion of ideas, stupor, giddiness, and so forth. The treatment consists in early, copious, and repeated depletions; to fix any specific quantity of blood to be withdrawn at one operation is ridiculous; we must be regulated by the effects produced; in most cases from 20 to 40 ounces will be found necessary. When the attack takes place in the 7th or 8th months, and is checked by the remedies employed, the woman rarely completes her full period of gestation; and labour comes on within a few days, or even in 24 hours. In the event of the disease not yielding, the only chance of saving the mother's and infant's lives, is in immediate delivery: but, as we have stated above, convulsions occasionally succeed the entire completion of labour; and from Dr. Ramsbotham's practice, it appears, that such cases are more intractable, and prove more frequently fatal, than when they occur previous to, or during labour. If they have commenced under these circumstances, and continued after delivery, the danger is much enhanced; but if, as often happens, they are checked, whenever the child is born, they seldom return; a quiet sleep succeeds, and the patient awakes refreshed and comfortable. As corroborative of these remarks, we present to our notice, a few of the very interesting examples which Dr. Ramsbotham has recorded.

"At four a. m. Friday, May 30th, 1817, a gentleman arrived at my house from a neighbouring village, to beg my immediate attendance upon the wife of a major in the army, in labour of her first child, and in a state of great danger. I was introduced to a young lady of a sprightly disposition, who had been attacked about an hour before my arrival with a convulsion-fit, during a common lingering labour, under the care of a respectable surgeon. Her labour had commenced on the evening of Wednesday; her attendant was then called, who remained in the house all night. It proceeded regularly, but slowly, throughout the day of Thursday; towards evening, the membranes broke; the pains afterwards became stronger and more frequent; but under great rigidity of all the soft parts. At this time, and for some hours thenceforward, the general appearances promised a happy termination. But this favourable prospect was, by and bye, overshadowed by the unexpected intervention of a convulsive paroxysm between three and four in the morning. The lady had hitherto been in excellent spirits, laughing and joking with her friends; when, just before the attack, she quaintly exclaimed, 'bless me, the room is studded with diamonds,' and instantly, her whole frame became violently convulsed. As soon as the necessary means could be procured, her attendant took away a quantity of blood from the arm, and sent to request my presence. I found this lady collected, and capable of answering my questions rationally; she complained of some unpleasant feelings in the head, yet not amounting to absolute pain; her pulse had undergone little alteration; the os uteri was opened to the size of a crown-piece, but was rigid and thick, with the head just entering the pelvis, pressing upon it; and the pains were returning at short intervals. I had not been long in the house, before another attack recurred, upon which I recommended the abstraction of more blood.

About seven, a. m. my friend and colleague, the late Dr. John Sims (who had been also summoned through the anxiety of the husband), arrived. After hearing the history of the case, and seeing the lady, he joined in opinion with me of the propriety of the loss of more blood. Not long after this third bleeding, we had the recurrence of another fit. While Dr. Sims was sitting by the side of the bed a little after nine, anxiously watching the lady, he witnessed the access and progress of another paroxysm, equally violent with any of the preceding ones. It was now evident, that the loss of blood, although upon the whole considerable, had neither prevented a return of the paroxysms, or diminished their violence; and that it could not be farther extended with safety; we therefore determined, that immediate delivery should be effected. About half-after nine, with the sanction of Dr. Sims, I perforated the head, and fixing the crotchet, began to make extraction; but the return of a paroxysm soon obliged me to desist during its continuance. After its cessation, my efforts were resumed to the release of the head. The uterus now contracted satisfactorily, and expelled the rest of the child with the placenta. The lady was afterwards lying in a senseless state; but after a short time, she appeared to be asleep; and remained quiet, and composed to the time of our departure at twelve o'clock. She had no return of convulsions after her delivery, but in due time recovered her health; and has borne several children since the preceding occurrence, without any tendency to a similar attack." 307.

The following is a case, which proved fatal, and in which the morbid appearances on dissection are detailed.

"At six, a. m. Saturday, January 26th, 1822, I was called to a poor woman in Fashion Street, Spitalfields, who had been attacked with convulsions. Her midwife had been summoned about two in the morning for some reason or other; but finding, to use her own expression, 'that there was no labour,' she did not remain with her patient. Some time after her departure, a convulsion-fit took place; the midwife was recalled; but before her return, five or six paroxysms had recurred; she then begged my assistance. I found the paroxysms violent, with short intervals, and a state of complete insensibility during those intervals. The os uteri was but little dilated, yet the liquor amnii was discharged, and the head was above the brim of the pelvis. I had a vein opened in each arm in my presence, and a large basin full of blood (I should think nearly two pounds) taken away from each orifice. Yet this free and liberal bleeding had no beneficial effect, either upon the violence, or the frequency of the paroxysms. After witnessing the recurrence of several more fits without any palliation, I determined upon immediate delivery by turning the child; but I had no trifling difficulties to overcome in attaining that object. During the act of delivery, and for a short time afterwards, the woman remained free from any return; she appeared relieved, and gave some hopes of recovery; but within an hour, another fit made its appearance, which she did not long survive.

The body was inspected the following day. After a most careful examination of the head, no positive breach of vessel could be detected. The blood-vessels of the pia-mater were beautifully injected with blood; and a section of the substance of the brain shewed more bloody points than usual; there was also a quantity of tinged serum in the ventricles. The vessels of the cerebellum were likewise unusually distended with blood. The viscera of the abdomen were generally healthy. The blood-vessels of the broad ligaments were empty, and seemed puffy and large. The uterine structure was flaccid; uncontracted; with a suffused redness at its back part; its internal surface had a natural appearance." 296.

In case CLXXXI the convulsions were accompanied with hæmatemesis, and proved very rapidly fatal. No time must be lost in the treatment of this formidable malady.

"On the 11th of March, 1796, a stout healthy young woman was delivered of her first child about six in the evening, having suffered comparatively little under a natural and easy labour, during which she made no particular complaint. I left the house after its completion, in the full expectation that she would do well. But about two hours after my departure, I was recalled in a hurry. I was then told that soon after I was gone, the woman was suddenly siezed with pain at the stomach, which was followed by vomiting and convulsions, and that after two or three paroxysms, she threw up a considerable quantity of blood. From the first appearance of the convulsions she became totally insensible and comatose, and continued in that state to the time of her death, which took place within fifteen hours after her delivery. Her situation precluded all aid from the exhibition of internal medicine, for none could be got into the stomach; the injection of clysters was recommended, but the unmanageableness of the patient rendered assistance from this source also nugatory. The ejection of blood from the stomach deterred me from the use of the lancet; indeed, I candidly confess, that at that early period of my professional career, I had not witnessed the beneficial effects of large bleeding; the practice of which was then scarcely established." 315.

Dr. Ramsbotham has observed that most of the cases of puerperal convulsions which he has seen, have occurred in hot sultry weather, especially when there was much electricity in the atmosphere. In one very alarming example, to which he was summoned during the raging of a violent thunderstorm, the lady had been very safely confined of her second child, for twelve hours, before any appearance of the fits, which so quickly succeeded each other, as scarcely to leave any interval. During the intermissions she lay in a state of total insensibility; and foamed at the mouth; the pupils were strongly contracted, and the pulse small, quick and confined. By large depletions of blood, her life was saved; and when she recovered, she had no recollection of the occurrence during her labour, although she appeared to be in perfect health at the time of delivery.

Does not this fact shew, says Dr. R., that there was even then a tendency to cerebral indisposition, which had escaped notice, and which was probably increased by the state of the atmosphere, and by the act of parturition? Before we quit the subject of convulsions, we must allude to a great omission in the remedies recommended by our author. There is one whose application is so easy, and whose energy is so potent, that we are inclined to rate its value as highly as we do that of blood-letting. In our practice its successful effects have been most satisfactorily established; and it is therefore with much confidence that we recommend it to the notice of Dr. Ramsbotham, and of our readers. The remedy to which we allude, is, the affusion of cold water on the vertex, so managed, that a minute stream is made to play uninterruptedly from a considerable height. By this simple but efficacious expedient, most cases of parturient, and infantile convulsions may be cured, provided resort is had to it at the commencement of the disease, before any serious vascular derangement occurs, or the system is so weakened by the severity of the paroxysms as to be unable to recover itself.

Upon the fourth section, which treats of labour with two or more children, we cannot afford much space to comment.

The proportion of twins appears to be about one case in 90 or 100 labours. When there is more than one fœtus in the womb, the woman has a less chance of completing the full period of gestation, than in a single conception: hence, miscarriages of twins is very common. In some cases, one of the ova loses its vitality, and is either retained in utero, or is aborted, while the other remains behind, and becomes fully formed.

As to the treatment of labours, in which there is more than one child, the following directions ought to be attended to. Immediately after the expulsion of the first, let the hand be placed on the belly of the mother, and if we have reason to suspect the presence of another, particular caution must be adopted, not to interfere with the protruded umbilical cord and placenta. Should uterine pains not come on in the course of an hour or two after the first birth, the second bag of membranes may be ruptured by the finger, or by a stilet; and if, notwithstanding the escape of the liquor amnii, the same tendency to inactivity should continue for some time longer, say two or three hours, we should then turn the child and extract it by the feet. Formerly these cases were left entirely to Nature's management, and were, in consequence, frequently attended with much protracted suffering, and sometimes followed by fatal issues. The following is a good example of such practice, in which the second child was left in utero for three days after the expulsion of the first.

"In the forenoon of Thursday, May 31st, 1821, my opinion was asked by a professional friend, upon the case of a woman, who had been delivered of a living child the day preceding at three, p. m. who had a second child in utero; and who, from the time of the expulsion of the first, had lost all further return of pain. I told him, that had I been attending the case, I would have delivered the second child before so much time had passed over; although it did not appear that the woman had hitherto suffered any inconvenience; but that under all the circumstances, I could not then advise immediate delivery. I recommended my friend to watch the case, and to await awhile longer the result. On the evening of Saturday, my assistance was requested; the woman still remaining undelivered of the second child. I then learnt, that on the morning of Friday, she had a violent shivering fit, which lasted a quarter of an hour; that no pains had returned from the expulsion of the first child till the middle of Saturday afternoon, when they became active; that the woman seemed nearly exhausted. The child's head was entering the pelvis, with the forehead to the pubes. It now seemed desirable, that this protracted labour should be speedily terminated; I therefore extracted the head by instrumental means, and the rest of the child was afterwards expelled by uterine action. After the delivery of the child, the woman began to flood very seriously; the uterus was at this time, to the external feel, flaccid and uncontracted. The hæmorrhage continuing, I presently found myself obliged to remove the double placenta by the introduction of the hand, a portion of which proved to be adherent. After delivery, there was little external discharge, but the woman complained of being very ill; indeed the uterus had again relaxed, and had become much more enlarged. On pressure, a quantity of fluid was thrown off, so that internal hæmorrhage was going on. I applied a strong degree of grasping pressure for some time, and left the woman in a hazardous state. She did not long survive my departure." 346.

In case CXCII, dangerous flooding had been occasioned by the medical attendant having attempted to withdraw the placenta of the first born, before he had ascertained the presence of a second child; and CXCIII, shews that fatal flooding may be induced by an unskilful extraction of the double placenta. At this part of his work, our author rather confusedly introduces some remarks on, and cases of dropsy of the membranes, and of ovarian dropsy; we shall first analyze the section on abortion, and class these other affections with the miscellaneous diseases which may all be sufficiently well grouped together. Miscarriages are more frequent in the females of the higher, than of the more humble classes of society, and take place more commonly before the period of quickening than after that occurrence: the period at which a pregnant woman is most disposed to this accident is between the 10th and 12th week of gestation. Dr. R. is inclined to attribute this to some imperfection in the change of fœtal circulation, which is usually about this time effected; for it is seldom that the placenta and funis can be detected before the end of the third month. The vulgar opinion that the fœtus does not possess vitality till the period of quickening, is absurdly enough recognised as true in criminal law; to the wilful destruction of the fruit of the womb after quickening, or in legal language, after the woman is quick with child, is awarded upon conviction, capital punishment; and to that destruction before this period, a less severe penalty. A medical man is often led to suspect the coming on of an abortion, by the absence, or disappearance of those feelings with which the early stages of pregnancy are commonly accompanied. The mammæ lose that increase of size and firmness, which they had lately acquired, and the morning sickness ceases. It often happens that when the lady has suddenly lost this troublesome companion, she is quite delighted, little aware that a most unfortunate change has taken place within her womb, of which there are at present no visible symptoms.

It is not unfrequent that the ovum dies, and yet is retained in the uterus for many weeks or even months, without exciting any general or local disturbance; the symptoms of pregnancy indeed cease, but the health may continue perfectly good. The younger the fœtus is at the time of its death, the sooner from that date will it be probably expelled; if it has reached the fifth or sixth month, it is often detained till the expiration of the usual term of gestation. The case may be suspected by the suspension of the common symptoms of progressive pregnancy. Occasionally, though it is comparatively a rare occurrence in a twin conception, one of the ova dies and is expelled, say about the third month, and the other one remains behind, and becomes a full grown child. Ladies are much puzzled and perplexed to account for this; the solution is obvious to a medical man. But the dead ovum is not always discharged; and at the end of the full period, the woman may be delivered of a perfect child, and of a dead ovum which had not passed the third month. A very singular fact observed in such cases, is, that this undeveloped fœtus sometimes exhibits no mark of putrefaction. The two placentæ are connected together, but one part of the mass is larger than the other. Contrasted with this curious phenomenon, it is not unfrequent, that if a single conception loses its vitality, and is afterwards detained in the uterus, even for a few days only, on expulsion it is found much decayed. In the treatment of miscarriages, it is to be kept in mind, that if a woman has repeatedly aborted at the same period of pregnancy, it is almost impossible to prevent the recurrence of a similar mishap; hence the especial necessity of unusual attention about the period at which it may be expected. Quietude of body and mind is indispensable, and all sexual intercourse must be avoided. After an abortion, it is not uncommon for a woman to complain of a feeling of bearing down; of a tendency to prolapsus uteri; this accident is more common after a premature, than after a regular labour; and arises from too soon quitting the recumbent posture. We subjoin one case.

"Several years ago, my attendance was requested upon Mrs. E. a young lady, who had been married some time, and who had had several miscarriages. During the course of the present pregnancy, upon which I was consulted, she had been repeatedly attacked with slight hæmorrhagic discharges, and pains threatening abortion, which had as repeatedly subsided. When she had attained the seventh month of pregnancy, however, she had uterine hæmorrhage succeeded by pains; these ultimately terminated, after a common labour, in the expulsion of a living child, but which did not long survive the birth. The uterus contracted well, and the placenta followed without trouble. When I called upon this lady twelve or fourteen hours after delivery, she seemed tolerably well, but she complained of pain, with a sense of pressure upon the external parts, as if something was about to pass through them. Not being satisfied with her account, I requested to make an examination, and immediately detected a substance of some kind in the vagina, pressing upon the perinæum, and external parts, of the size of an orange; behind which I could readily pass my finger. Introducing too fingers behind this mass, which readily gave way, I hooked out a perfect ovum; that is, 'an embryo within its membranes entire, at little more than the third month of gestation.' Upon opening the membranes, there was a male fœtus (the sex just discoverable), of about that age; perfect as far as it went, and without the least disposition to putrefaction.

The mystery respecting the repeated tendencies to abortion, was now satisfactorily cleared up. The lady had conceived of twins, and went on well to the third month, when one ovum lost its vitality. For the purpose of ridding itself of this dead animal substance, the uterus had instituted certain operations, which were counteracted by management." 402.

### EXTRA-UTERINE PREGNANCY.

The female often does not suspect any imperfection or irregularity, till she has completed the full period; if she then experiences any pain, the doctor and nurse are summoned, in expectation of labour; but when the uterus is examined, it is found to be little developed; a solid, hard tumour is felt in the belly, more inclined to one side than to the other, and rather tender on pressure; the increase of size is not uniform over the whole abdomen, nor to the usual extent of common pregnancy; but the nature of the case is seldom clearly made out, till the process of the destruction of the fœtus commences, and a discharge of flesh, bones, &c. outwardly, takes place. In some rare instances the misplaced ovum has remained very stationary for a length of time, interfering little with the health, and producing no annoyance, except such as arises from pressure on the neighbouring parts. The woman has even become pregnant meanwhile in the regular way, and been delivered of a living child. The usual progress, however, of extra-uterine conceptions is the establisment of an adhesive inflammation between the cyst and the abdominal parietes, intestines, or body of the uterus; ulceration follows, and the fœtus is discharged piecemeal; the mother's health generally sinks under the protracted suffering. Should the contents suddenly escape into the cavity of the belly, death may very rapidly ensue. A curious variety of misplaced conception is when the ovum is arrested in that portion of the fallopian tube which perforates the uterine structure; for in this case, the whole of the uterus is temporarily developed, as under regular pregnancy; but that portion in which the fœtus is detained becomes more enlarged than the rest of the organ. The progress of the symptoms is nearly the same as in other examples of this singular aberration. Upon dissection the uterus presents a strange appearance; one part being little developed, with the os tincæ attached to it; the other portion extensively enlarged, with an obvious rupture connected with the abdominal cavity, but without any opening into the hollow of the womb. Many cases which have been supposed to be rupture of the uterus are of this kind. We shall analyse the six cases of extra-uterine pregnancy narrated by Dr. Ramsbotham, stating the most prominent features of each.

In case CCI. the woman observed that the enlargement of the belly was chiefly confined to the left side; she suffered much from sickness, pain in the left side, and most obstinate costiveness, which resisted ordinary purgatives; she felt the motion of the child; the mammæ were enlarged. The motions ceased in the sixth month; labour threatened to come on several times, and, after much anxiety and suffering, she passed per rectum the thigh-bone of a child, about 12 months from the commencement of her pregnancy. She died of protracted diarrhœa; the catamenia had returned. On dissection, a cyst was found full of bones and putrid matter, communicating with the arch of the colon.

In case CCII. the swelling was chiefly on the right side, and felt hard and circumscribed; bowels very much confined; catamenia suspended; mammæ at first full and painful, afterwards flaccid. Nearly two years after the commencement of pregnancy, an intractable diarrhœa supervened; and, after it had continued for four months, fœtal bones were discharged from the bowels; this woman recovered. The menstrual discharge was quite regular from the seventh or eighth month after conception, till within a month or two before the fragments of the child were first voided.

In case CCIII. the lady considered that she became pregnant at Christmas, 1818. The swelling of the belly was chiefly on the left side, which became hard and solid to the finger; she felt the motion of the child. The period of gestation having elapsed, it was believed that she had not been in the family-way at all. A large tumor was still perceptible on the left side, where it continued stationary; she occasionally felt severe pain in it; and the bowels were most obstinately confined. In August 1823, she was confined of a living child at the full time. In March, 1824, she passed by stool the thighbone of a fœtus; and soon afterwards many more bones were discharged. The issue of the case is not known.

In case CCIV. the patient conceived in December, 1827; quickened in May following; and the movements of the child were afterwards so violent occasionally, as to be seen through her dress. In August, she felt pains which she thought were labour-pains; at this period, she felt the motion of the child strong and lively; but soon afterwards it ceased entirely. A watery discharge at first, and then a fetid one began to issue from the vagina; and a bone was passed not long before Dr. R. was consulted, in the following November. At that time she was suffering from severe pains in the belly, and the bowels were very costive; there was a large, hard, circumscribed and tender tumour in the belly, inclining rather to the right side of it. In the beginning of 1829, a swelling formed at the umbilicus; it broke and emitted much offensive fluid, mixed with fæcal matters; in February, some of the phalanges of the fingers passed through the opening; similar discharges escaped by the rectum, and also by the vagina. She gradually sunk, and died in May. On examining the abdomen, the cyst was found to communicate not only with the abscess outwardly, but also with the intestinal canal and the uterus. The uterus, both ovaries, and fallopian tubes were entire and healthy. We may, therefore, suppose the above to have been a ventral pregnancy.

Case CCV. proved rapidly fatal. A young lady had symptoms of miscarriage three months after marriage; she complained also of a constant pain in the lower part of the belly, which was tender, but little swelled. While combing her hair and preparing for bed, after passing a better day than usual, she was suddenly seized with an alarming faintness and pain, and in a few hours expired, with all the symptoms of internal hæmorrhage. On dissection a quantity of fluid blood, mixed with coagula, was found in the abdomen; it had escaped from the right fallopian tube, which was formed into a cyst, containing an embryo ten or twelve weeks old. The uterus was of the ordinary size.

Case CCVI. is quite similar to the preceding one.

### Dropsy of the Membranes.

The cause which usually excites an encreased deposition of the liquor amnii is quite mysterious; the health is usually not interfered with thereby, except in consequence of the inconvenience produced by the size, weight, or pressure of the accumulated fluid. Nor does the uterine system appear to be much injured by it; for, after it has been discharged, the womb contracts, and speedily regains its accustomed functions. The quantity of liquor amnii amounts in some instances to several gallons, by which the uterine parietes are excessively extended and attenuated; indeed to such a degree occasionally, as to render fluctuation under the hand distinctly perceptible: hence the frequent mistakes of ascites, for this affection of the womb, or of its contents. A correct diagnosis is of the highest importance, and may generally be arrived at, if the following signs are attended to. In dropsy of the membranes, there are present at first the symptoms of pregnancy; the belly encreases rapidly and somewhat suddenly in size within a short time; and the patient seems otherwise in good health. The reverse of all this usually holds true in reference to ascites; and besides, this disease for the most part occurs at a period of life, when conception is out of the question. Let it however be remembered, that both forms of dropsy may exist contemporaneously—such a case requires great sagacity and most inquisitive attention to detect. As however there will be always more or less doubt, it ought to be a rule in practice, that the complete term of pregnancy be suffered to pass over, before any decisive means of relief be resorted to.

An example will best illustrate the importance of this observation.

"A lady, thirty-six years of age, the mother of several children, after the common symptoms of incipient pregnancy, became, soon after the fourth month, unusually large for that period of gestation; at the same time complaining occasionally of a violent pain in the side of the abdomen, which would attack her suddenly, and as suddenly disappear. There were at this time a quick pulse, a dry tongue, with scantiness of urine, which was thick and turbid, and not more in quantity than half a pint in twenty-four hours. The swelling of the belly increased rapidly until fluctuation was perceptible; which at first was obscure, but afterwards was more evident. Her respiration became quick and laboured; general emaciation took place; she appeared in great distress, yet the legs were not œdematous. The general symptoms now so nearly resembled those of ascites, that it became doubtful whether she was pregnant or not. About the middle of

January, 1812, a physician was consulted, who pronounced the case to be ascites, and prescribed diuretics. Soon afterwards she was seen by another physician, who was disposed to think the case one of ovarian dropsy; he prescribed purgatives, and mercurial ointment with camphor to be rubbed upon the abdomen. She was, after no long lapse of time, seen by an eminent surgeon, who considered the case to be ascites, and proposed tapping. Under all the circumstances of the case, the operation was thought desirable; yet, as there had been some doubts about pregnancy, it was previously judged expedient to enquire into the state of the uterus. A vaginal examination was accordingly made; upon which the os uteri was found a little open, and through it, a tense bag of membranes was detected; all further proceedings were therefore for the present suspended. Shortly after this examination, when the lady had reached something more than the fifth month of pregnancy, her usual attendant was called, in consequence of her being attacked with pains, which she considered to be labour-pains; at this time she was of an immense size. The pains presently became of a more decided character; the os uteri was considerably dilated, with a tense bag of membranes protruding through it. He passed his finger through the bag, and a deluge of fluid immediately rushed out to the quantity of several gallons, flooding the room, and putting into requisition mops and cloths, to sop it up; upon this discharge the size of the belly disappeared, and the uterus began to contract more actively. Notwithstanding this sudden evacuation, the woman merely complained of faintness. The labour-pains went on gradually to the expulsion of a dead fœtus under a breech-presentation, apparently between the fifth and sixth month. The state of the uterine tumour induced a suspicion that there was a second child; this proved to be the fact. Uterine action was presently re-established, and another dead child was expelled. When the after-births were withdrawn, the uterus was found to be well contracted; the woman afterwards went on well; and recovered her former health under common management in the usual time." 351.

Case CXCVI. exemplifies well the co-existence of ascites and pregnancy.

"In the latter part of the year 1819, Mrs. W. in Cripplegate, became in a family way, and in the early part of her pregnancy had symptoms of ascites. This complaint induced a suspicion that she might be mistaken as to the state of pregnancy; she consulted an eminent accoucheur, who pronounced her to be pregnant. The process of gestation, and the dropsical symptoms, kept pace with each other to Sunday afternoon, August 6th, 1820, when she was delivered of a healthy living child, after an easy and quick labour, even before her accoucheur could arrive at the house. On the Wednesday following, I was desired to visit her: at this time, her abdomen was enormously swelled, with an evident fluctuation; her breathing was rapid and difficult; the pulse was small and quick; she complained of constant pain in the belly; and seemed to be in the greatest distress. These symptoms had been rapidly aggravated since her delivery, especially the abdominal extension. Seeing no chance of even temporary relief but in the evacuation of the fluid, I requested that a surgeon might be called in, who introduced a trocar, and drew off between four and five gallons of limpid serum. The next day she seemed to be considerably relieved; but in a few days it was too obvious, that the abdominal cavity was again rapidly filling; in this way she lingered on for a short time, and gradually sunk. The woman was in her forty-second year, and had borne sixteen living children." 354.

Dr. R. reports also a case in which pregnancy and ovarian dropsy existed at the same time; the possibility therefore of such an occurrence it is of much moment to recollect.

With respect to the origin, or mode of formation of what are termed

"moles," or "false conceptions," our author is induced to believe that they frequently arise from a blighted ovum, which, though retaining its adhesion to the uterus, is in time converted into a solid inorganized mass, totally unlike any fœtal structure.

The reader should remember, that pregnancy is often very closely simulated by such growths; and also by the accumulation of hydatids within the womb. Although it is abundantly easy in general to ascertain the existence of pregnancy, yet cases happen to every medical man, which not a little puzzle and perplex both him and his patient. It is not an uncommon occurrence for women, who even have had children, to suppose themselves to be in the family way, when in fact they are not so. The catamenia indeed cease, and in time the abdomen enlarges; our patient attributes this to impregnation. She gives way in her dress; indulges any whim of appetite for articles of food and drink, and neglects her bowels; flatus is accumulated, and the rumbling is mistaken for the movement of a child; the distension increases; and perhaps some pains come on at the period of the expected accouchement; but upon a vaginal examination, the uterus is found to be unimpregnated. Now it is sometimes no easy task to ascertain the particular cause of the mistake; it may be from a diseased ovarium, or from a deposit of fat in the omentum, or under the abdominal muscles; or from a loaded and unhealthy state of the alimentary canal; or from a combination of all these occurrences. It is important to remark, that although the belly is large, yet the hand cannot detect any thing like a uterine tumor, except perhaps in ovarian enlargements; and if we examine per vaginam, we shall generally arrive at the truth. When the medical man is satisfied of the mistake of his patient, he will find that a few doses of calomel and purgatives, followed by tonics, the use of a bandage, and bathing, will speedily dissipate all the symptoms. To shew however the vexatious uncertainty of such cases, we shall give a condensed report of one, which is detailed by Dr. Ramsbotham.

In December, 1826, he visited a lady, mother of a large family, and supposed to be in the 6th month of pregnancy; there was an excessive enlargement of the uterine tumor, with a painful extension of the abdominal parietes, so that the belly was as prominent as at the full period; it was resisting on pressure, and did not convey any feeling of fluctuation: what was singular, it had chiefly taken place within the preceding fortnight;—a vaginal examination gave little satisfactory knowledge; the os uteri was slightly open, thin and relaxed, the cervix was extended and scarcely perceptible. Under the impression of pregnancy, the great distension could only be referred to dropsy of the liquor amnii; a temporising plan of treatment was recommended. This patient continued in a state of feverish disturbance for two months, when parturient pains commenced, the os uteri opened, the membranes protruded, but no part of the child could be felt. The bag burst, and a rush of liquor amnii instantly followed; soon after a child between the fifth and sixth month was expelled.

In December, 1827, Dr. R. was again consulted; the lady was supposed to be three months gone with child, yet the belly was as large as at the end of the 9th. The uterine tumor was distinctly felt, large, firm, and resistant, and very tender on pressure, especially at one point towards the right ileum, where the pain was exquisite. A constant discharge, sometimes sanguineous, at other times serous, with a few coagula, but inodorous, had escaped from the vagina for the preceding five weeks. The symptoms being altogether so similar to those which existed on the former occasion, were at once attributed to the same cause, especially as the catamenia had been regular for several months before the period at which she supposed that conception took place, and as afterwards a progressive, although a very unusual increase of size followed. The cervix uteri was elongated and thickened; the os tincæ was soft, flaccid, and a little open, so as to admit the passage of the finger about half an inch; yet nothing like the bag of membranes was perceptible; and the body of the viscus was obviously much enlarged. The treatment adopted was merely palliative. Four days after this date, Dr. R. again saw her; the abdominal distension was rapidly encreasing, and the general health so much disturbed, that considerable apprehensions of a fatal issue were entertained. Under the belief of pregnancy, a catheter was carried high up into the cavity of the womb, but no fluid escaped. Next day the patient was much distressed by pains every now and then returning; she appeared quite exhausted, and died before noon.

*Sectio Cadaveris.* The abdomen was tumid and soft, having lost much of its former firmness; when the parietes were divided, a quantity of an offensive gas escaped. The uterus was of the size which it has in the 6th month of pregnancy; flabby, and had

> "A red appearance on its external surface, yet not indicative of inflammatory action. The stomach and intestines were distended by gas; the other viscera had a healthy aspect. Upon removing the uterus out of the pelvis, the os uteri was open and flaccid, and within it was seen a tinge of dark redness, which was afterwards found to pervade the whole of its inner surface. Upon dividing the uterine structure, its parietes were thickened and enlarged, as under pregnancy, but no appearance of a fœtus could be detected. The cavity merely contained a fibrous mass, about the size of a large egg, loosely attached to the posterior surface, and entangling within its substance, a number of small coagula. This mass was surrounded by a quantity of bloody purulent fluid, in which were floating other small coagula. The whole of the internal surface of the viscus was in a state of disease, which on a close inspection put on the appearance of granulating eminences; in some points, especially near the cervix uteri, its structure was destroyed by the ulcerative process, almost to the peritoneal covering. The uterine cavity would have contained a body equal in size to the infantile head at full time. The ovaria, the fallopian tubes, and the vagina had a healthy appearance." 427.

Retroversio Uteri usually occurs a short time before quickening, when the dilated and dilating organ nearly fills up the cavity of the pelvis, and is about to emerge therefrom into that of the abdomen. It may generally be traced to arise from an over-distended bladder, aided probably by some momentary occurrence, giving to it increased effect. Hence in treating this malady, the chief indication is to evacuate the bladder, and to prevent it from becoming again distended. Should, however, the uterus not right itself in a few days, an artificial attempt to replace it must be made, by introducing two or more fingers of the left hand within the rectum, for the purpose of pushing up the fundus, while at the same time, with the other hand in the vagina we draw down the cervix. The patient, during the operation, ought to rest on her elbows and knees, her body forming an inclined plane. If repeated attempts to effect this prove fruitless, it may be deemed necessary to induce abortion, or at least to discharge the liquor amnii; and the less scruple may be felt in doing this, as we know that miscarriage, at all events, or premature labour, will sooner or later ensue. Considerable difficulty may be experienced in the operation, in consequence of the displacement of the parts; and Dr. R. tells us that, in the only instance in which he performed it, he succeeded by introducing an elastic bougie; on the second following day, the fœtus and placenta were expelled. Seven months after this period, the lady became again pregnant; and in the third month the uterus was retroverted a second time; but fortunately soon righted itself, when the bladder was emptied. She went on to her full time, and was delivered of a perfectly grown child.

It is possible that other diseases may be mistaken for retroverted uterus, even by accoucheurs of great tact and experience; and we therefore cannot do better than extract, from this most valuable repertory of cases, one which illustrates the truth of our remark; but are necessitated to abridge the details. In March, 1820, Dr. R. first visited the patient. She had been married several years, but had never been pregnant. The belly was enlarged, and the bowels obstinately confined, and the urine very scanty for the previous three weeks. Purgatives produced no effect; and the dysuria became more distressing, as the water kept constantly dribbling away. On introducing the catheter, an operation which had been very improperly neglected by her former medical attendants, a wash-hand basin full of urine was drawn off; the hand now introduced into the vagina found a large firm tumor entirely filling up the pelvis, and pressing upon the rectum; it could not be pushed aside by any pressure. The bladder was afterwards relieved daily by the catheter. Another distinguished accoucher now joined Dr. R. in consultation; both considered the case to be retroversio uteri; and it was agreed first to attempt to replace the organ, and should that fail, to rupture the membranes; but in neither operation did they succeed. A third physician was called in, and his opinion coincided with that of the others;—again the membranes were attempted to be ruptured, but the trial was ineffective. The belly was now much swelled; the bowels so costive as to resist all medicines, either by mouth or in injections; in a few days she died.

*Dissection.* On dividing the abdominal parietes a large cyst was discovered, partly covered by omentum, to which it adhered, and likewise to the peritoneum lining the muscles, and to the fundus of the bladder. It was vascular, and fluctuated when pressed upon. Upon puncturing it several quarts of serous fluid were discharged. The urinary bladder was empty, and apparently quite healthy in structure; the uterus was also healthy, unimpregnated, and found lying upon a smaller cyst, which was of such a size, as to fill up the whole cavity of the pelvis, and to contain probably two or three pints of fluid. These two cysts proved to be the left and right ovaria, the uterus being situated horizontally between the two, with its orifice above the pubis. The distension of the abdomen had been caused by the dropsical ovary of the left side; and the right one, by its firm adhesions within the pelvis, and the consequent pressure upon the bladder and rectum, had produced all the singular symptoms observed during life.

### Polypus of the Uterus.

Whenever a female, of mature years, has a much increased menstrual discharge, and suffers from occasional hæmorrhages, and a constant draining of a serous, mucous, or purulent discharge in the intervals; when she has pains in the back, with a sense of pressure, or dragging downwards, it is the duty of a medical man to suspect a polypus, and to examine the state of the uterus.

A tuberculated state of the internal surface of this organ will indeed give rise to most of the symptoms of polypus; these tubercles may sometimes be felt, by introducing the finger into the os uteri; they have not a narrow arch and base like polypi, but are of the same breadth throughout; and they very slowly increase in size. It is well known that similar growths are frequently found upon the external surface of the womb, and are denominated the fleshy, or white tubercles. Sometimes these tumors acquire the size of an orange, and induce much inconvenience or suffering, if situated between the uterus and rectum. In one case mentioned by Dr. R. all the symptoms of diseased rectum were present; and it was at length discovered that ulceration between the gut and tumor had taken place.

Polypus of the uterus occasionally, though rarely, is met with in unmarried females; as is exemplified in Case CCXIII.

The inverted uterus has been mistaken for polypus, and been attempted to be removed. Dr. R. candidly avows an error of this sort which he once committed. The day after delivery, a fleshy tumor, of the size of a large pear, was protruded suddenly through the external parts, and there it remained; the woman was in little pain; and to appearance was doing quite well. Some forcible efforts to pull the mass away, under the idea that it was a false conception, had been made by her medical attendant. It was now gently returned into the vagina, and perfect quietude enjoined. The tumor did not again descend; and she soon recovered, having during all the time suckled her child. Some months afterwards she again became pregnant, at which Dr. R. was much surprised, as he thought it improbable that she should conceive if the uterus was inverted; however, in due time she was confined. During the labour Dr. R. had felt a substance of a considerable size in the vagina, and above this the head of the child presenting; as the pains became strong, the tumor was first expelled and then the head. On minutely examining the protruded substance it was found attached to the fore part of the os uteri by a broad and vascular base. For the present, it was cautiously replaced, and the patient did well, and was able to nurse her child. It was observed that, as the uterus became contracted, the polypus decreased in size. It was afterwards removed by ligature.

Case CCXVI. is a good illustration of the perplexing embarrassments which sometimes await the obstetrical practitioner, in the diagnosis of tumors felt in the vagina. The lady had, for a long time, suffered from repeated hæmorrhages; her health was beginning to give way. On examination, a large firm and round mass, occupying nearly the entire cavity of the pelvis, was detected; the finger could not be carried well round it, nor reach the os uteri. Several other swellings could be felt by the hand in the hypogastrium, on the external surface of the uterus. A double ligature was applied on the supposed polypus; but very disagreeable symptoms appeared on the second day, and it was, therefore, removed; but the patient speedily became worse, and she died three weeks after the operation. A post-mortem inquiry was not allowed; and we are, therefore, left in utter doubt as to the true nature of the disease. In such a case, however, we should not follow the example of Dr. Ramsbotham, but be satisfied with mere palliative treatment, till the os uteri can be distinctly felt. It is better, moreover, that a patient should die of a disease, than of the means employed for its removal; and the fatal result was very evidently hurried in the example we have just quoted. By leeching, or even, perhaps, by an incision into the mass, its size may be so diminished as to permit a more satisfactory examination, without which it is imprudent and unsafe to resort to its extirpation.

Appended to the present volume, are the detailed reports of three cases of that most formidable accident, "rupture of the uterus," in which the patients recovered perfectly. It is very singular that, at the period of publication of the first volume of these Observations, in 1820, every case which Dr. Ramsbotham had then seen had, sooner or later, proved fatal; and that, within 12 months afterwards, he witnessed three cases of complete recovery. In two of these, he turned the child himself. We shall briefly analyze them, for the benefit of our readers. In the first, the woman was attended by a midwife, who dispatched a note to the Doctor with these words—"Pray come directly, for the afterbirth is come, and the child is gone." The placenta was found lying in the bed, without any hæmorrhage, and hanging out of the parts by the funis. On passing the finger, no part of the child could be felt; the head, which had previously been well down in the pelvis, had retreated quite out of reach; through the abdominal parietes, the child could be felt by the hand. Without loss of time, the funis was divided and the hand introduced; by following the child into the cavity of the belly, and laying hold of the feet, it was extracted without much difficulty. After delivery, she still complained much of tenderness about the navel, at which part she experienced a most acute pain, immediately before the placenta was expelled. The after-treatment consisted of opiates, leeching, purgatives, and emollient enemata. Dangerous fever supervened; the urine was passed involuntarily; the belly was exceedingly tense and uneasy; but from this state she recovered; and the report, six weeks after the delivery, announces that her only inconvenience arises from the involuntary discharge of urine. A lump or tumour, which she had noticed at the lower part of the belly for some time past, was subsiding. Six weeks after this date she menstruated. In the following year she again became pregnant, and died of flooding when six or seven months advanced. The following are the appearances on dissection.

"There were several bands or strings of membranous structure just above the pubes; but there were no marks of adhesion of the peritoneal lining of the cavity to the uterus, as I expected to meet with. On dividing into the uterine cavity, the placenta was seen partially separated from its attachment not far from the anterior part of the cervix uteri, to the size of nearly the palm of the hand. The child was now taken out, and the uterus, with the bladder, were carefully examined by a minute inspection. The vagina was considerably contracted, and had an extra opening into the uterus, and another into the bladder. The membrane of the vagina was not carried over the os uteri in the usual manner, but terminated in a valve-like structure; within which was seen the os uteri beautifully studded with its peculiar dark-coloured glands, and unopened.

On dissecting away the cellular membrane and adipose substance from the outside of the fore-part of the cervix uteri, a line or scar was distinctly perceptible; around this part the uterine structure was thickened and considerably contracted. The placenta had been attached internally not far from this thickening; and it appeared to me, that the rigidity or want of extensile power in the uterine structure around this part, had caused that separation of the placental mass, which had induced the fatal hæmorrhage. The ovaria and fallopian tubes were perfectly healthy." 492.

*Case* 2. The head had already began to bear down on the perineum; suddenly the pains left her. and she screamed out "I am sick—something has bursted in my belly;" and she begged to have a pillow put under her belly, for she could not endure the weight of the child. Violent retching and hiccup came on; the face was of a dark livid colour, and the eyes stared wildly. The head could not now be felt in the vagina—the labour-pains had quite ceased, and she vomited a dark brown fluid. The hand was introduced; and, passing by the head of the child, which was lying just above the brim of the pelvis, both feet were seized, and delivery speedily accomplished. The placenta, lying in contact with the intestines, was then withdrawn. Although the convolutions of the bowels were distinctly felt, the operator was not sensible, either in bringing down the feet, or in removing the after-birth, that his hand passed through any rent of the uterus; it seemed at once to pass into the cavity of the abdomen. Opiates and brandy were given; on the following day blood was drawn, in consequence of pain and tension of the belly. She recovered so well, that, after the expiration of a fortnight, she was free from all complaint except debility. Menstruation returned in two or three months after the accident, and not long afterwards she became pregnant again. It was deemed prudent not to allow gestation to exceed the seventh month; and had not premature labour come on at that period, it was determined to have brought it on. It proved to be an arm-presentation; but no difficulty occurred in turning the child, and the woman recovered without any untoward circumstance.

In the third case, the symptoms were nearly the same as in the preceding; and this woman, too, speedily proved again in the family way. Pregnancy had been allowed to go on till the full period; and, when labour commenced, the pains were strong and expulsive, but the head did not descend. The conjugate diameter of the brim was found to be scarcely three inches; it was, therefore, thought advisable to deliver by perforating the head. In a subsequent pregnancy, the brim of the pelvis was found to be still more contracted, and recourse was, therefore, immediately had to delivery by the crotchet. She recovered, also, from this confinement.

We shall close our review of these observations, by transcribing an interesting case of what may be denominated "uterine apoplexy," or rupture from congestion; and, as it is a specimen of a rare disease, we shall give the details at length.

"I was engaged to attend a young married lady in West Smithfield, who expected to be confined of her first child, the first or second week in January, 1812. She went into the immediate neighbourhood of her own house, to join a party of friends on the evening of New-Year's-Day, being at that time apparently in perfect health; and after she had been among them a few hours, in full enjoyment of the hilarity of the evening, she suddenly complained of being very ill. With great difficulty she was got up stairs, and was seated in an easy nursing chair; presently she fell lifeless on the floor!! The people about her supposed her to be in a fainting fit; a neighbouring medical man was called, who, in the first instance, thought the lady in a state of syncope; but presently pronounced her to be dead!! Some time was lost in the confusion which prevailed in the house; but by and bye a messenger was dispatched for me. I arrived at the house a little after midnight; at that time, the lady had been lifeless at least an hour. Notwithstanding, I would gladly have removed the child by the Cæsarean section, but her friends would not consent to that operation.

Leave was obtained to inspect the body the next day, yet only on condition that a near relative should be present. On dividing the abdominal parietes, the gravid uterus presented itself to view, but very different in its aspect from that which is generally met with. The whole of the fore-part of the fundus, and some portion of the back part of the uterus was completely black, not unlike that appearance upon the skin of a delicate woman, after the infliction of a severe blow. The fallopian tubes were turgid and black; the ovaries were of a natural size, but they had a striated or speckled appearance, somewhat like mottled soap. Upon making an incision into the peritonæal coat of the uterus at its back part, where the black or suffused appearance was the most obvious, fluid blood freely followed the knife. The placenta was attached at the fore-part of the body of the uterus throughout its entire extent, and the child was presenting naturally; the internal uterine surface seemed healthy. The stomach, the intestinal canal, and the other abdominal viscera had the usual healthy appearance. The heart and the large blood-vessels were healthy and sound; within the pericardium was contained a small quantity of serous fluid; the right lung was a little diseased, with trifling adhesions to the pleura costalis; the left lung was healthy. The head was not allowed to be examined.

The above appearances led me to suspect that some large vessel had given way within the uterine structure, the contents of which had been effused into the cellular tissue under the peritonæal coat; producing a state in the gravid uterus similar to that of the brain under effusion beneath its meninges." 507.

Having thus given a very extended and comprehensive analysis of Dr. Ramsbotham's book, we deem it quite unnecessary to expatiate on its merits; our best eulogy is the number of pages which we have devoted to its consideration, for the judgment of a reviewer, like the appetite of a guest, may generally be known from the use which he makes of the viands before him:—We have largely explained to our readers the bill of fare; we have done more—for they have here set before them most of the choicest dishes, and we have given them to taste of the most racy of the wines; but still our advice is, "go unto the feast yourselves," and ye will be largely fed and abundantly satisfied.

Dr. Ramsbotham's observations meet with our very cordial approbation; they are the faithful records of a very ample practice, and coming thus, with all the authority and sanction of multiplied experience and well matured reflection, they cannot fail to exercise a salutary and instructive influence on all those engaged in obstetrical pursuits. To such as are at a distance from the advice and co-operation of their more advanced brethren, we especially commend this volume; they will find it useful as a work of reference, to guide and direct them. It will serve the purpose of a consulting physician, for under almost every circumstance, they will here meet with a corresponding and illustrative example; such is a great advantage of a well-described and judiciously-selected catalogue of cases upon any particular theme; it is like a cabinet of natural history, to which the student refers either to ascertain the name and position of a specimen, with which he is unacquainted, or to refresh and fortify his memory ; and so it is with a good case-book, which the young physician may consult to guide and to confirm his practice. Moreover, the information acquired from the reports of actual cases, is always much more impressively stamped upon the mind of the reader than that from descriptive narration, for he is amused and interested while he is instructed. On this account, let the present volume be admitted into the library of every practical man: there is not, perhaps, much strictly novel matter in it, but yet there is much that is important; it is a plain unvarnished picture of what the author has witnessed and done by the bedside of his patients, and of what almost every medical man may be called upon to witness and to do ; and, after the study of it, what shall we say of those conceited puppies, or doting fools, who presume to depreciate and disparage the utility and importance of midwifery ; or what shall we think of medical colleges, who recognize not its study among the necessary qualifications of their candidates.

One word to our author before we leave :—In a second edition, which we hope will soon be required, let the language and style be well revised and corrected ; although manly and vigorous upon the whole, it is frequently rather prolix, quaint, and obscure ; such tautologies as "let it be ever kept in mind, and let it never be overlooked;" and "unsuccessfully attempted and repeatedly attempted;" or such expressions as "falling into labour," "in the morning of a certain night!" "the area of a diameter ! !" "a child lively and likely for life," and many others, which we have neither leisure nor time to enumerate, ought to be carefully expunged or corrected. Let not elegance of diction and propriety of style, in medical writings, be undervalued by any one; for, as a picture is set off to advantage by a handsome and becoming frame, so is the materiel of a book by the beauty and grace of the language in which it is conveyed. Many of the reports of the cases may also be very advantageously abridged and condensed ; the little histories or notices of particular times, events, engagements, and so forth, affixed so often at the commencement of them, should be lopped off. It is not, however, agreeable to look out for blemishes in a pretty face, or in a lovely landscape ; neither is it to our taste to expatiate any longer on the minor defects of a good, a valuable, and an instructive work, like that which Dr. Ramsbotham has presented to our review.

